# Profiling Proteins in the Hypothalamus and Hippocampus of a Rat Model of Premenstrual Syndrome Irritability

**DOI:** 10.1155/2017/6537230

**Published:** 2017-01-31

**Authors:** Mingqi Qiao, Peng Sun, Yang Wang, Sheng Wei, Xia Wei, Chunhong Song, Fushun Wang, Jibiao Wu

**Affiliations:** ^1^Key Laboratory of Traditional Chinese Medicine for Classical Theory, Ministry of Education, Shandong University of Traditional Chinese Medicine, Jinan 250355, China; ^2^Laboratory of Ethnopharmacology, Institute of Integrated Traditional Chinese and Western Medicine, Xiangya Hospital, Central South University, Changsha 410008, China; ^3^Technical Office of Pharmacology, Shandong Institute for Food and Drug Control, Jinan 250351, China; ^4^College of Psychology, Nanjing University of Chinese Medicine, Nanjing 210023, China

## Abstract

Premenstrual syndrome (PMS) refers to several physical and mental symptoms (such as irritability) commonly encountered in clinical gynaecology. The incidence of PMS has been increasing, attracting greater attention from medical fields. However, PMS pathogenesis remains unclear. This study employed two-dimensional gel electrophoresis (2DE) for proteomic map analysis of the hypothalamus and hippocampus of rat models of premenstrual syndrome (PMS) irritability. Matrix-assisted laser desorption/ionisation time of flight mass spectroscopy (MALDI-TOF-MS) was used to identify proteins possibly related with PMS irritability. Baixiangdan, a traditional Chinese medicine effective against PMS irritability, was used in the rat model to study putative target proteins of this medicine. The hypothalamus and hippocampus of each group modelling PMS displayed the following features: decreased expression of Ulip2, tubulin beta chain 15, *α* actin, and interleukin 1 receptor accessory protein; increased expression of kappa-B motif-binding phosphoprotein; decreased expression of hydrolase at the end of ubiquitin carboxy, albumin, and aldolase protein; and increased expression of M2 pyruvate kinase, panthenol-cytochrome C reductase core protein I, and calcium-binding protein. Contrasting with previous studies, the current study identified new proteins related to PMS irritability. Our findings contribute to understanding the pathogenesis of PMS irritability and could provide a reference point for further studies.

## 1. Introduction

Premenstrual syndrome (PMS) is a symptom complex that periodically appears during the latter half of the menstrual cycle, accompanied by physical, mental, and behavioural changes [1–3]. It is most commonly seen among women of childbearing age, between 30 and 40 years. Recent literature shows that PMS is a common syndrome among young women and is closely linked with mood, body, and behaviour. PMS has a large coverage of pathogenesis and quite a high morbidity at the same time [4]. The etiology and pathogenesis of PMS are primary aims of basic research in this area; targeted drug research in traditional Chinese and Western medicine has also become a current hotspot.

PMS irritability is a major type of PMS, with features of premenstrual dysphoria, irritability, breast distending pain, and abdominal distension or pain, followed secondarily by insomnia, dreamful sleep, headache, gastral cavity distension, belching, acid regurgitation, premature menstruation, reduced sexual desire, distaste for sexual life, attention-deficit disorder, fatigue, bulimia, diet partiality, lumbago, tendency to tears, and so forth [5] Baixiangdan, a traditional Chinese prescription created by Cyperus (*Cyperus rotundus* L.), Common Peony (*Paeonia lactiflora* Pall.), and Peony bark (*Paeonia suffruticosa* Andr.) (Supplementary Material available online at https://doi.org/10.1155/2017/6537230, Figure S1), have been shown to effectively relieve symptoms of PMS irritability in clinical trials and animal experiments. Studies revealed that the main active compounds of Baixiangdan were paeoniflorin, paeonol, and alpha-cyperone [6–8], which might have antipyretic, anti-inflammatory, analgesic, and neuroprotective functions [9, 10]. The pathogenesis of PMS irritability, which is very complex, involves the mental, neural, and internal secretion systems. So far, no one has clarified the mechanism therein; hence, no studies have reported will-dominant PMS irritability prevention. Previous studies have revealed that the occurrence of PMS could be related to functional disorders of the hippocampus and the hypothalamus [11–13]. Consequently, the hippocampus and the hypothalamus are the anatomical regions that have been most closely studied regarding the pathogenesis of PMS.

There are changes in the mRNA expression of central monoamine neurotransmitter receptors including 5HT1A, 5HT2A [14], and GABAA [15] receptors and ER*α*, ER*β*, PGR, and central steroidal hormones receptors, in the limbic system of a macaque model with PMS irritability [16]. Aberrant expression of receptor genes could affect their function and further lead to abnormities in the expression of the key protein(s) in downstream signal pathways regulated by these receptors, potentially representing an important link in triggering the disease.

The present study used a rat model of PMS irritability for proteomic screening based on a previous microarray study [16], analysing the differences in expression of the central organization protein linked with this disease in the rat model and a control group, through two-dimensional gel electrophoresis (2DE) and matrix-assisted laser desorption ionisation time of flight mass spectrometry (MALDI-TOF-MS). In addition, the relationship between the protein and the incidence of PMS irritability is discussed below, to explore its mechanism of action. The Baixiangdan capsule was also administered to the rat to determine its possible target protein. The present findings are expected to lay the technical foundation for the proteomic study of this medicine.

## 2. Materials and Methods

### 2.1. Laboratory Animals and Ethics Statement

Healthy female SPF Wistar rats weighing 160–180 g were selected. They had ad libitum access to water and food. The feeding room temperature was 24 ± 1°C and the relative humidity was 50 ± 10%. Animals were provided by the Laboratory Animal Center of Shandong Traditional Chinese Medicine University, license number: SCXK (LU) 2011-0003. Laboratory animals were provided care according to “*The Care and Use of Laboratory Animals*” by the Laboratory Animal Center of Shandong University of Traditional Chinese Medicine.

### 2.2. Drugs

The Baixiangdan capsule was procured from Xiuzheng Pharmaceutical Group Company Limited. It was jointly developed by Qingdao Haichuan Innovative Biological and Natural Medicine Research Center with the batch number: 2007L05105.

### 2.3. Determination of the Oestrous Cycle

All grouped rats were weighed, recorded, and marked with picric acid, and their oestrous cycle was determined using the vaginal smear (Supplementary Material, Figure S2) microscopic examination method [17, 18]. On a proestrus vaginal smear, epithelial cell nuclei and a few keratinocytes are present, while on an oestrous vaginal smear, enucleate keratinocytes and a few epithelial cells are present. On a postoestrous vaginal smear, leukocytes, keratinocytes, and epithelial cell nuclei are found, and on an anoestrous vaginal smear, there are large numbers of leukocytes, few epithelial cells, and myxocytes. Rats with short estrous cycles (about 3 days) were included in the following experiments.

### 2.4. Generation of a PMS Irritability Rat Model and Baixiangdan Capsule Treated PMS Irritability Model

Following vaginal smearing [8, 19], 30 rats in the postoestrous period were divided randomly into 3 groups with 10 rats in each: namely, the control group, the group modelling PMS irritability, and the group treated with the Baixiangdan capsule. The PMS irritability group and the group treated with Baixiangdan were connected to ST-A digital pulse stimulators. The stimulation conditions were as follows: voltage 2700–3300 V, pulse width 0.3 s, pulse separation 5 minutes during the daytime and 10 minutes during the night, and continuous stimulation for 5 days. Five-day stimulation covers a dioestrus and a round of the oestrous cycle. As for the group treated with the Baixiangdan capsule, Baixiangdan was intragastrically administered to animals at 0900 hours each day and lasted for 5 days when modelling stimulation was in progress. Drug dosage for the rats was 1 mg/100 g, once each day for 5 days, representing almost 8 times the dose typically taken by human patients.

### 2.5. Movement and Exploratory Behaviour (Open-Field Test)

The field box model number was XR-XZ301 from Shanghai Xinran with dimensions of 50 × 50 × 50 cm^3^. Data acquisition software was the animal behaviour trace analysis system XR-Xmaze. The specific operations were the following: the operator held the 1/3 part of the rat's tail and gently placed the rat in the center of the field box, following which the observer recorded the rat's movement trace, modification time, time spent standing in the central grid, number of excrement particles [20–22], and so forth.

To eliminate rat odour, the field box was cleaned with 75% ethanol after each rat was tested. After drying, data acquisition for the next rat was conducted. The grabbing action in this experiment was as gentle as possible to eliminate artificial intervention.

### 2.6. Attack Behaviour Test

The attack behaviour test [22] was conducted between 1430 and 1730 hours inside the rat housing environment. After day and night inversion, this timeframe was the exciting period for the rats. The specific operations performed were as follows: rats were placed in the cage; an invading rat (which had been spayed) was then placed into the cage after 15 minutes of adaptation, for 10 minutes. Composite aggression was calculated using the following formula: Composite aggression = [(number of attacks) + 0.2 × (attack duration) + (number of bites) + 0.2 × (on-top duration) + (piloerection)].

### 2.7. Rat Cerebral Tissues Sampling

After decapitation, the operator dissected out the hippocampus and hypothalamus on an ultra-clean platform and placed them in liquid nitrogen immediately for quick cooling. After 30 minutes, they were then stored at −70°C. All operation instruments, watch glasses, and Eppendorf tubes in this experiment were sterilized in an autoclave. The ultra-clean bench surface was wiped with ethanol above 75% and exposed to UV light, to prevent pollution.

### 2.8. Protein Extraction and Quantification

For protein extracting and quantifying methods, we referred to the literature [23]. The hippocampi and hypothalami were dissected out from the rats; lysate buffer [self-prepared (7 M urea, 2 M thiocarbamide, 4% CHAPS, 1% DTT, 1 mM EDTA, and 40 mM Tris pH 7.4)] was added in a ratio of 150–170 mg : 500 *µ*l (sample : lysate); further, cocktail protease inhibitor (Sigma-Aldrich, St. Louis, MO) was added for oscillating and blowing; freeze-thawing was conducted using liquid nitrogen, which was followed by ultrasonication; DNase I (Sigma-Aldrich) and RNase A (Sigma-Aldrich) were added; centrifugation was conducted at 4°C with 14,000 rpm for 25–30 min; supernatant was then packaged and stored at −80°C, and protein levels were quantified.

### 2.9. Two-Dimensional Gel Electrophoresis

Two-dimensional gel electrophoresis (2DE) was performed as previously described [24, 25]. Samples were subjected to isoelectric focusing in a 17-cm immobilized pH gradient pH 3–10 (Biorad Inc., Berkeley, CA) overnight, using the Protean Isoelectric Focusing Cell (Biorad). The second-dimensional separation of focused samples on the gel strips was performed through sodium dodecyl sulphate-polyacrylamide electrophoresis (SDS-PAGE) using 13% linear polyacrylamide gels. A previously described silver staining method was used to develop the 2DE gels (Heukeshoven & Dernick, 1988). All samples were analysed in duplicate.

### 2.10. Image Analysis

Before scanning, bubbles and other impurities were eliminated to ensure there were no water droplets in the photic zone; ImageMaster 2D. v3.01 analytic software was used for protein spot detection. With spot detection guidance, optimal detection results were determined by adjusting sensitivity, and the operator and background factor, to achieve automatic detection. After detection, manual editing, such as the addition, deletion, and splitting of spots was needed for unidentified, wrongly identified, and vaguely identified spots, respectively, caused by congestion due to phosphorylation or other reasons. After manual editing, ImageMaster automatically calculated the number of protein spots. The in-gel digestion method [26] was performed to extract peptide fragments.

### 2.11. Protein Identification with the MALDI-TOF-MS Technique

The peptide fragment mixture extracted using trypsin needs to be desalted; hence, a 50% (v/v) acetonitrile-containing suction nozzle was used to balance by 0.1% trifluoroacetic acid (TFA) twice; a 0.1% TFA solution was used to dissolve and clean the extracted samples; samples were placed in 5 *µ*l cyano cinnamic acid (CCA) saturated matrix solution; 2 ul treated samples were applied on the target spot on the mass spectroscopy plate. Spectrometric detection was conducted in positive ion reflection: N_2_ laser source; wave length, 337 nm; flight tube length, 3 m; accelerating voltage, 20 kV; reflection voltage, 23 kV; and matrix CCA [8]. The matrix peak and the trypsin autolysis peak were considered as the interior standard.

### 2.12. Database Retrieval

The obtained peptide fragments fingerprint spectrum was retrieved in the Swiss-Prot database (http://www.matrixscience.com).

### 2.13. Statistical Analysis

Graphpad Prism 5.0 statistical drawing software (Graphpad software Inc., CA) was used to analyse the experimental data. The statistical analysis for the behavioural assays was performed using one-way ANOVA (*n* = 10). All data are shown as the mean ± SD, with the significance level set at *P* < 0.05.

## 3. Results and Discussions

### 3.1. Identification of the Rat Model with PMS Irritability and the Effect of the Baixiangdan Capsule

The Baixiangdan capsule is a patented Chinese medicine developed in accordance with the prescription of traditional Chinese medicine. This traditional medicine has been considered effective in treating PMS symptoms, like premenstrual vexation, irritability, headache and distension, breast distending pain, insomnia, dreamful sleep, abdominal distension and pain, and premature menstruation. In addition, the pharmacology and pharmaceutical studies have demonstrated that this medicine has specific active ingredients that can ease PMS [8, 27, 28]. In the current study, emotional stimulation, combined with interference to sleeping, eating, and drinking, was used to generate the rat model in the postoestrous period [7, 8, 27, 29], though such a modelling method was not accepted by all sciences. Further, we administered the Baixiangdan capsule to intervene in the rat model and compared the control group with the model group to identify PMS irritability-related proteins using proteomics.

**Figure 1:**
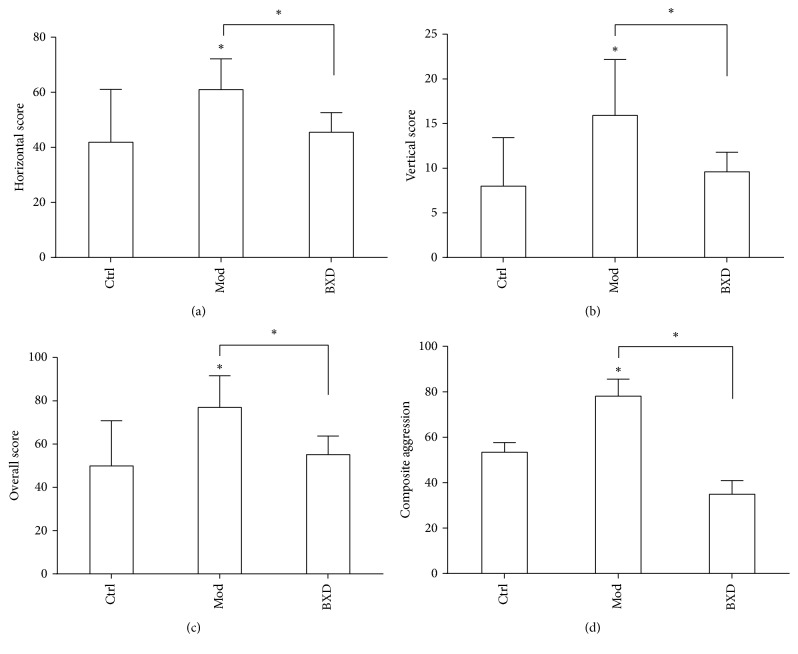
Behavioural assays. (a) Horizontal score in the open-field test, representing the rat's excitability. (b) Vertical score in the open-field test, representing the rat's exploratory behaviour. (c) Overall score in the open-field test. (d) Score in the attack behaviour test. The following groups were analysed: (1) the control/normal group (Ctrl), (2) the PMS irritability model group (Mod), and (3) the group administered with the Baixiangdan capsule (BXD). ^*∗*^*P* < 0.05.

The field test can be used to evaluate animal excitement, cognition, and interest in the outside world. Two scores are computed on two axes: the horizontal score, reflecting the animal's excitement, and the vertical score, reflecting the animal's tendency to explore an uncertain outside world [30–32]. The total score for the open-field test reflects the exploratory behaviour and excitability of the animals. The results indicate that the horizontal scores, the vertical scores and the total open-field scores of rats in PMS irritability group (Mod) are all significantly increased (Figures 1(a)–1(c),* P* < 0.05), compared with those of the normal group (Ctrl). Combining these results with the macroscopic observation of the rats (for behaviours such as staring angrily, fur standing, chasing, or biting), the prepared model could be considered a successful model of PMS, though the electro-stressing method of modelling was controversial. Compared with the scores of the model group (Mod), the horizontal, vertical, and total open-field scores of the rats in the Baixiangdan capsule medication group (BXD) are all significantly decreased (Figures 1(a)–1(c),* P* < 0.05), indicating that the Chinese herbal compound, Baixiangdan, has had a sedative and antianxiety function.

Results for the test of aggressive behaviours are similar to those of the open-field test. Compared with that of the normal group (Ctrl), the score for the rats' aggressive behaviours in the PMS irritability group (Mod) is significantly increased (Figure 1(d),* P* < 0.05). Compared with that of the model group (Mod), the score for the rats' aggressive behaviour in the Baixiangdan capsule medication group (BXD) is significantly decreased (Figure 1(d),* P* < 0.05). For each of the above open-field and aggressive behaviour tests, one experiment was conducted before modelling each group, to act as baseline; there were no statistically significant differences between the groups (not shown,* P* > 0.05).

### 3.2. Image Analysis of 2DE Serum Protein Profiling

**Figure 2:**
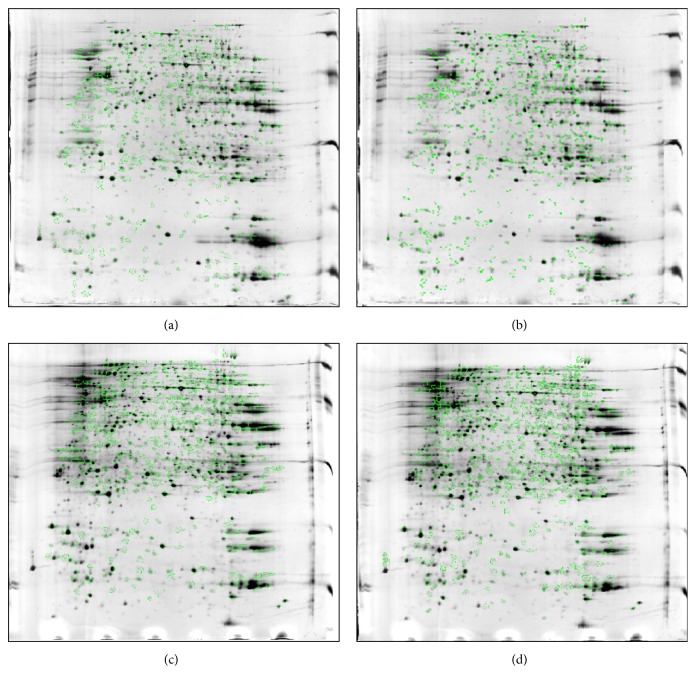
Two-dimensional gel electropherograms obtained from hypothalamus and hippocampi tissues, analysed in pairs. (a) Image overlay of the hypothalamus samples from the control group and the model group. (b) Image overlay of the hypothalamus samples from the control group and the group administered with Baixiangdan. (c) Image overlay of the hippocampi samples from the control group and the model group. (d) Image overlay of the hypothalamus samples from the control group and the group administered with Baixiangdan.

High-resolution serum proteome profiles were obtained from the normal controls (Ctrl), the PMS irritability model rats (Model), and the PMS irritability rats given the Baixiangdan capsule (BXD) through 2DE separation and silver staining of the hypothalami and hippocampi lysates. We analysed the two-dimensional gel electropherograms obtained from the hypothalami and hippocampi tissues in pairs, and obtained 4 overlapping ones: Ctrl versus. Mod hypothalamus (Figure 2(a), 61% matched), Ctrl versus. BXD hypothalamus (Figure 2(b), 72.5% matched), Ctrl versus. Mod hippocampi (Figure 2(c), 56% matched) and Ctrl versus. BXD hippocampi (Figure 2(d), 63.5% matched). In general, after treatment with the Baixiangdan capsule, protein expression approached normal levels or displayed that tendency (Figure 2). The proteins in the rat hippocampus and hypothalamus that were differentially expressed for rats with PMS irritability, control rats, and rats given the Baixiangdan capsule were analysed, and there were 22 types of proteins that underwent changes of expression: upregulation of 6 types and downregulation of 16 types.

### 3.3. Identification of Expressed Biomarkers Using Mass Spectrometry

After in-gel tryptic digestion, MALDI-TOF-MS was used to obtain the peptide mass fingerprint spectrum, with the aid of the website (http://www.matrixscience.com) for identification. Among the 22 proteins, 11 were identified (Table 1); these were Ulip2 protein, tubulin beta chain 15, *α* actin, aldolase, M2 pyruvate kinase, panthenol-cytochrome C reductase core protein I, hydrolase at the end of ubiquitin carboxy, albumin, interleukin 1 receptor accessory protein, hemoglobin *α* chain, kappa-B motif-binding phosphoprotein, and calcium-binding protein. When compared with the normal group, the model group displayed the following features of hippocampus organization: decreased level of Ulip2 protein, tubulin beta chain 15, *α* actin, and interleukin 1 receptor accessory protein and increased level of kappa-B motif-binding phosphoprotein. The hypothalamus organization of the model group, when compared with that of the normal group, displayed the following features: decreased expression of hydrolase at the end of ubiquitin carboxy, albumin and aldolase, and increased expression of M2 pyruvate kinase, panthenol-cytochrome C reductase core protein I and calcium-binding protein. For more information, please check Table 1 for the protein name, molecular weight, the isoelectric point, the protein identification score, and the peptide fragment matching rate.

A preliminary study was conducted on any differences between the normal group and the PMS irritability group in terms of protein expression in the hippocampus and hypothalamus. There were 22 types of proteins with differential expression, 11 of which were subjected to mass spectrum identification. Since the enzymes may not digest all the peptide fragments and some hydrophobic and large peptide fragments may get lost, the coverage rate of peptide fragments in this experiment was 22–76%. The identified proteins can be divided into the following types in terms of function: (1) cytoskeletal protein; (2) intermediary metabolic enzyme; (3) ubiquitin path and protein enzyme related protein; (4) signal path protein; (5) transcription, shearing, and extending related protein; and (6) calcium-binding protein. However, this present study did not identify any protein expression changes in central monoamine neurotransmitter receptor and central steroid hormone receptor in the PMS irritability model, which is not consistent with previous research at the mRNA level [16] probably because of the hydrophobic property of these proteins. For example, in enzymolysis, peptide fragments with a hydrophobic property and large molecular weight may get lost, leading to the loss of important information. Another issue is that it is difficult to explain how the particular proteins identified in this study cause PMS irritability, based on our understanding of their functions. The two aforementioned points constitute the main limitations of this study. Nonetheless, this study presents new findings that have not previously been reported, thereby contributing to research on PMS and providing the foundation for further exploration of the aetiology and pathogenesis of PMS. For example, we intend to further investigate the calcium-binding protein, signal path protein, and transcription-related protein, with the administration of more precisely selected chemical compounds in place of Baixiangdan, to more clearly identify the regulatory mechanism. So far, gratifying achievements have been made and future studies would soon follow.

## Supplementary Material

Figure S1: Herbal medicines in Baixiangdan. Figure S2 :Vaginal smears in different estrous cycles.

## Figures and Tables

**Table 1:** Between-group differences in the expression of proteins in the rat hippocampus and hypothalamus.

Accession	Mr		Cal. PI	Peptides		Sequence coverage
	Theor	Cal				
A25113	49905	2	4.79	21	41	60%
AAA37166	16758	5	5.29	6	11	39%
XP-347150	15275	6	8.45	6	6	58%
A54143	51010	10	5.19	9	12	22%
AAB93667	57744	12	7.15	20	35	44%
XP-217267	52815	13	5.57	8	23	25%
AAH17646	31353	14	4.94	11	17	45%
NP-599153	68674	17	6.09	20	21	44%
NP-036629	39259	18	6.67	13	27	40%
AAH39177	24822	20	5.14	14	20	76%
XP-485555	49754	22	4.73	17	44	39%

## Abbreviations

2DE: Two-dimensional gel electrophoresis
SDS-PAGE: Sodium dodecyl sulphate-polyacrylamide electrophoresis
PMS: Premenstrual syndrome
MALDI-TOF-MS: Matrix-assisted laser desorption ionisation time of flight mass spectrometry
Ctrl: Normal control group
Mod: PMS irritability model group
BXD: PMS irritability rats given Baixiangdan capsule.
