# Supplementary material for: Profiling Proteins in the Hypothalamus and Hippocampus of a Rat Model of Premenstrual Syndrome Irritability
Source: Neural Plast. 2017 Jan 31;2017:6537230. doi: 10.1155/2017/6537230 (PMC5306999; doi:10.1155/2017/6537230)

*Figure S1 Herbal medicines in Baixiangdan.*


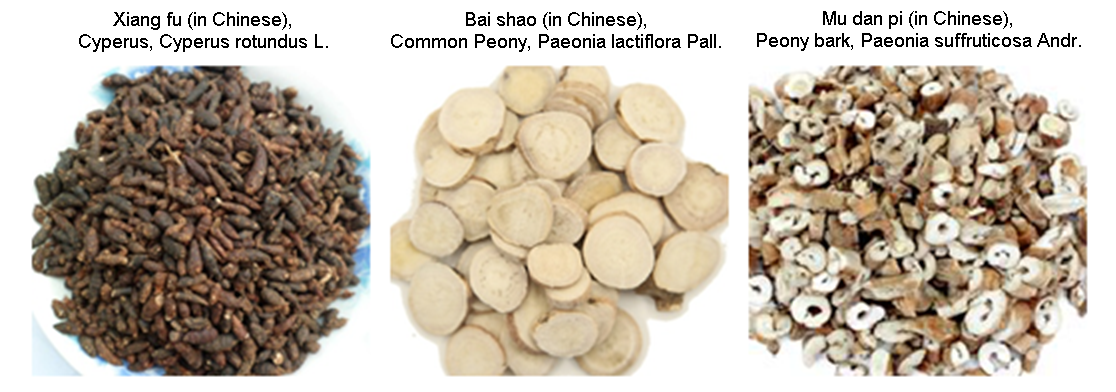


*Figure S2 Microscopic observation of the vaginal smear. Scale bar: 1 mm.*


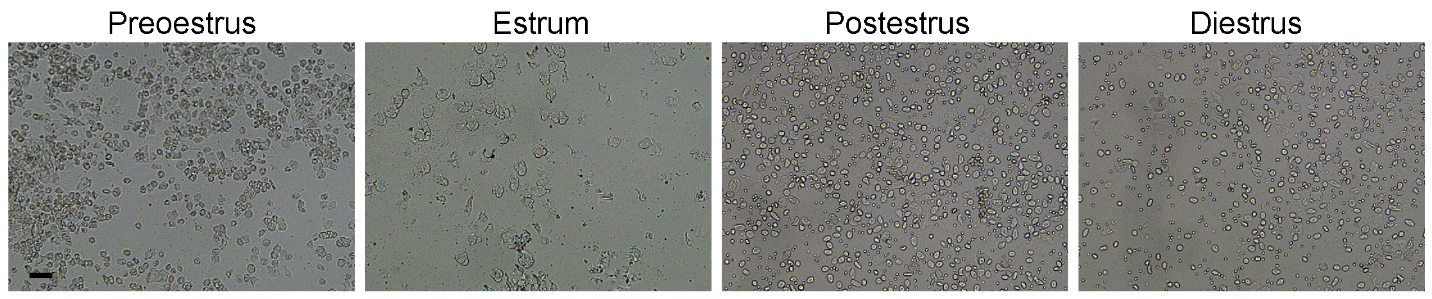

Supplement: Supplementary file 1 — Figure S1: Herbal medicines in Baixiangdan. Figure S2 :Vaginal smears in different estrous cycles. [file 6537230.f1.docx]
